# Shared decision-making between health care providers and patients at a tertiary hospital diabetic Clinic in Tanzania

**DOI:** 10.1186/s12913-020-06041-4

**Published:** 2021-01-04

**Authors:** Osward Vedasto, Baraka Morris, Francis F. Furia

**Affiliations:** 1 Department of Bioethics and Health Professionalism, School of Public Health and Social Sciences, Muhimbili University of Health and Allied Sciences, Dar es Salaam, Tanzania; 2 Department of Paediatrics and Child Health, School of Medicine, Muhimbili University of Health and Allied Sciences, Dar es Salaam, Tanzania

**Keywords:** Shared decision-making, Diabetic patients, Muhimbili national hospital, Decision aids

## Abstract

**Background:**

Patients’ participation in decision making regarding their treatment is defined in ethical, legal and human rights standards in the provision of care that concerns health providers and the entire community. This study was conducted to document experiences of patients and health care providers on shared decision making.

**Methods:**

This study employed a phenomenological study design using in-depth interview technique. Study participants were diabetic patients visiting the clinic and healthcare providers working at Muhimbili National Hospital. Data was collected using the semi-structured interview guide with open-ended questions using an audio digital recorder. Content analysis method was used during analysis whereby categories were reached through the process of coding assisted by Nvivo 12 software.

**Results:**

Participants in this study expressed the role of shared decision-making in the care of patients with diabetes, with report of engagement of patients by health care providers in making treatment decisions. Participants reported no use of decision-making aids; however, health education tools were reported by participants to be used for educating patients. Limited time, patient beliefs and literacy were documented as barriers of effective engagement of patients in decision making by their healthcare providers.

**Conclusion:**

Engagement of patients in decision-making was noted in this study as experienced by participants of this study. Time, patient beliefs and patient literacy were documented as barriers for patients engagement, therefore diabetic clinic at Muhimbili National Hospital need to devise mechanisms for ensuring patients involvement in treatment decisions.

**Supplementary Information:**

The online version contains supplementary material available at 10.1186/s12913-020-06041-4.

## Background

Diabetes mellitus is one of the chronic diseases affecting about 463 million people globally.,The community prevalence of diabetes mellitus in Tanzania is 5.7% and more than 500,000 people are estimated to be living with Diabetes [1, 2].. Effective management of Diabetes calls for collaborative efforts between health care providers, patients and patients’ families [3]. Meaningful collaboration can only be realized with engagement of patients and their families in decision making regarding treatment choices and long-term care. This will ensure autonomy and self-management which play important role in the management of chronic conditions [3–5]. Shared decision making empowers patients with ability of voicing their preferences, participation in self-management and adherence to chosen treatment plans [6]. Unfortunately, shared decision making is poorly implemented, researched and documented in low-middle income countries specifically in sub-Saharan region [7–10]. Poor implementation of shared decision making is attributed to healthcare providers paternalistic attitude, patients’ passivity, patients’ trust in their healthcare providers, lack of applicability due to limited supporting resources (patients aids, infrastructure, human resource capacity) .

Globally, there has been a major shift in the provision of health services that focuses on patient centered approach. This approach makes patients the key drivers of care in order to achieve long term treatment goals., [11, 12]. This shift mandates a move from paternalism (health provider centered) to shared decision making with a fair balance of power and sharing of necessary information between patients and healthcare providers [9, 10, 13–15].

There are limited studies on shared decision-making conducted in sub-Saharan Africa. More specifically, in Tanzania very little is known on the experience of implementation of shared decision making in healthcare settings. Therefore, this study was conducted at Muhimbili National Hospital to document the experience of healthcare providers and diabetic patients in shared decision making.

## Methods

### Design and sample

A qualitative phenomenological study design was used to document the experience of healthcare providers and diabetic patients in shared decision making in the diabetic clinic at Muhimbili National Hospital (MNH). This design was appropriate for documenting the experience of participants on shared decision-making at the clinic [16, 17].. The planned sample size was eighteen participants (4 medical specialists, 5 registered nurses, and 9 diabetic patients). However, only eleven participants were recruited based on inclusion criteria for healthcare providers and saturation for patients. These eleven participants that were selected purposively; this included four healthcare providers (2 medical specialists and 2 registered nurses) who had been working at the clinic for at least one year were recruited and seven diabetic patients were recruited as they had regularly visited and received healthcare at the clinic for more than six months, were adult and mentally fit for interview.. Muhimbili National Hospital (MNH) is the largest referral hospital in Tanzania and it serves as the secondary referral facility for three regional hospitals in Dar es Salaam city (Mwananyamala, Temeke and Amana) and it is the teaching hospital for Muhimbili University of Health and Allied Sciences (MUHAS). Diabetic Clinic at MNH is under the Department of internal medicine; the clinic is staffed with 3 nurses, and 4 medical specialists. On average two medical specialists attend 40–60 on each clinic day. The clinic days are Tuesday, Wednesday and Thursday, which usually run from 10.00 am to 4.00 pm.

During the face-to-face in-depth interview, participants described their experiences of shared decision making, as they knew it. An interview guide with prepared questions and probes was used to ask respondents on: general understanding of their participation in shared decision-making, factors that influence diabetic patient’s participation in shared decision-making, significance of shared decision making, the use of decision-making aids, challenges encountered in executing shared decision-making, and strategies to improve the execution of shared decision making. The investigators in this study included OV (holding Bachelor of Art and Philosophy) who was the principal investigator and at the time of this study masters’ student in the Department of Bioethics, BM (BSc Nursing, Masters of Bioethics) who is a faculty in the Department of Bioethics and FFF (MD) who is a faculty in the department of paediatrics at MUHAS.

### Data collection

Following Institutional Review Board Approval at the Muhimbili University of Health and Allied Sciences (MUHAS), participants’ written informed consent was obtained. The face-to-face individual in-depth interviews among participants who consented to participate in the study commenced from ***January to May 2019***. Two interview guides, one for health care providers (supplementary material 1) and one for patients (supplementary material 2) were developed in English and translated to Swahili were pilot tested in the same clinic and used in this study; interviews were conducted in Swahili (National language for Tanzania). All study participants were given the freedom to choose a language (English or Swahili), which they will be comfortable for the interview. Coincidentally, all participants were confortable to be interviewed in Swahili. Hence, all interviews took place in Swahili then it was translated to English after the transcription of the interview. The average duration of each interview was 30 min.

The Interviews were conducted in a dedicated room at the clinic, at the time of interview only participant and the interviewer were present to ensure privacy. Interviews were audiotaped after getting permission from the research subject and notes of important cues were taken. The interview guide provided open-ended questions that were important and focused to solicit participants’ descriptions of their experiences in shared decision-making. Probing was done in areas that needed more clarification or elaboration. The end of data collection was marked by the saturation of information. Saturation of information was determined by reviewing each audio and short field notes taken after each interview to get the new information. The point of saturation was reached at the seventh patient interview as no new information emerged, for health care providers all (2 nurses and 2 medical specialist) were interviewed.

### Data analysis

Inductive Content analysis was used to analyze the data.. After data collection the audio recorded interviews were transcribed verbatim and later translated from Swahili to English. The translated transcripts were then checked for grammatical errors and translated back to Swahili for consistency check. The translated transcripts were familiarized through multiple reading after which coding plan was developed from the text and study objectives. After getting the general sense of the data, similar or related codes were grouped together to form categories. Coding was carried out by principal investigator and discussed the formulated categories with the two other investigators. Once consensus was reached the adopted categories were aligned in relation to study objectives to generate themes. Themes were assistance NVivo12 software program.. Locating text segments and assigning them to a particular category defined the creation of themes. The creation and definition of themes was done interactively, and all identified themes were evaluated and checked by other investigators for consistence and redundancy.

## Results

### Demographic characteristics of participants

Healthcare providers (HCPs) comprised of 2 female registered nurses (in their early 30s) and 2 male medical specialists (one 30s and one early 40s) .

Three female and four male patients who were married and aged between 46 and 76 years were included in this study.. Education level of these patients ranged from standard seven to advanced diploma. Two female patients were housewives and one was self-employed while two male patients were retired civil servants, one was a driver and one was a businessman.

### Themes obtained in the study

Three themes were obtained in relation to the practices and challenges of participation in shared decision-making between diabetic patients and healthcare providers at Muhimbili National Hospital (MNH). These themes are role of shared decision-making, decision aids and barriers to shared decision-making.

### Role of shared decision making

Patients reported to have been engaged in conversations with healthcare providers regarding decision made on their treatments but there was no clear decision making reported from patients. Healthcare providers reported that they always engage patients with diabetes in decision-making regarding screening and treatment options. They reported that at the diabetes clinic all decisions involve physicians, nurses and patients. Following examples prove this:

“*But in my experience, I engage properly my patients through conversations*” (#02 D).

“*Okay, a patient participates when he comes to look for service and doctor’s explanation on that particular condition or problem.*” (#03 D).

“... *I have to agree with my patients that retinopathy screening is voluntary. I don’t force a patient to screen because we have to discuss and agree with each other*” (#01 N).

Healthcare providers reported that they like to involve patients in shared decision making (SDM) because it helps them to determine the patients’ understanding of a disease, to understand their chief complains, to adhere to HCPs’ advice on treatment, to actively participate in self-management at home and it enhances a good relationship between HCPs and patients. One of the doctor’s responses was,

“*When a patient participates it helps me to know his/her chief complaint and so that I can decide on the appropriate course of action to be taken. You know diabetic patients are under self-management therefore for them it is very important.*” (#02 D).

Most diabetic patients reported that they are engaged in shared decision-making and that it is very important. Participation in decision-making helps health care providers to understand patients’ preferences in the treatment options. Also, it helps health care providers to determine the type of drugs that are suitable for the patient. The following examples proved patient’s participation and its importance.

“*It is important as the doctor will know which medication or drug works for me and that which doesn’t. Also, it will help him to know drugs that a patient likes and those he or she doesn’t like and why*.” (#07 P).

Some few participants reported minimal or partial participation in decision-making. They reported that they do not participate because sometimes providers make decisions on their own. They also reported that only healthcare providers have the right and responsibility of deciding what is best for a patient because they are experts and patients have only to abide on what health care provider decide and plan. They said that:

“*I don’t make any decision, when I visit clinic like today I just inform my doctor about my condition and he decides what tests or drugs are suitable for me*” (#06 P).

Some participants expressed fearcribed, citing fear of uncovering adverse reactions which may affect adherence, this participant said:

*“Unh … it is better I’m not engaged … (Laughing) You know you may be told that the drugs you take are not safe so I, like any human being may say let me stop using them.*” (# 06P).

### Decision-making aids

Participants provided no report of existence of decision-making aids in this study. Healthcare providers acknowledged that they use different materials including pictures, charts, leaflets and plates, for diabetic health education to make sure patients understand appropriate diet, complications as well as treatment options. Patients are sometimes told to use other sources including Internet. However, these materials were not prepared for supporting decision-making. They said that:

“*Okay, in most cases we use them and mind you that when patient comes here, he/she must read them. The important thing that makes you to use them is that some patients prefer to be taught using pictures …*” (#01 N).

Healthcare providers expressed concerns that education materials that may facilitate decision-making were not sufficient at the clinic. These materials are usually donated to the clinic, and neither healthcare providers nor the hospital management were involved in their preparation. They said:

“*Leaflets are available, but they are scarce. They are sometimes available and the other times not as we at Muhimbili are not producing them therefore we have to request them from donors. .*” (#02 D).

### Barriers to shared decision-making

Some of the factors reported by participants as barriers to shared decision making included beliefs and values, time, and educational level.

Some patients indicated that they do not participate in a shared decision-making because their beliefs and values do not allow them to. They believe that healthcare providers must be respected and considered the same as local witch doctors. This makes a patient to be resistant to be engaged in decision-making and leads to partial or no shared decision-making. One patient said:

“*According to our traditional values you can’t question a witchdoctor, but you have only to comply on what he directs like bringing him a cock or whatever. This applies to our professional doctors as well.*” (#08 P).

Healthcare providers and patients with diabetes held that there is shortage of time for active engagement in decision making between health care providers and patients, this was attributed to high provider-patient ratio for each clinic as well as multiple tasks of providers which limit their presence in the clinic. Respondent said:

“*I might have a patient who need to stay with me during consultation for at least for 15 minutes but I have to squeeze the time because of the long queue of patients who are waiting outside.”* (#03 D).

*“The time to talk to a physician satisfactorily is not enough... sometimes you find there is one doctor while the number of patients waiting is big and there is t patients overcrowding …*.” (#05 P).

Some healthcare providers claimed that it is difficult to engage a patient with low level of education in decision-making. They insisted that patients with low education do not understand things quickly and easily, making patients in this category not to be engaged in decision-making. Therefore, low education level of some patients was used to justify one-sided decision. For example, a doctor indicated that:

“*… So you have to consider the education level of your patients at times as it may be difficult for them to understand … is easy to just make decision and give treatment.”* (#02 D).

## Discussion

This study was conducted to document the experience of healthcare providers and patients at Muhimbili National hospital diabetic clinic, given the limited information regarding involvement of patients in decision making in sub-Saharan African and Tanzania in particular, findings of this study provide unique perspective on this subject and provides basis for future studies to further explore other aspects of interaction between patients and provider in provision of services.

Shared decision making plays an important role in supporting self-management of patients with chronic conditions for better outcome [13, 18, 19]. Participants in this study expressed their experience and views on shared decision-making. Likewise, participants expressed the need for decision-making aids and the important role of shared decision making to ensure patients autonomy and safeguard patient rights. These findings are consistent with findings from Ghana and Waweru et al. from Uganda, which have pointed out the need for decision-making among patients with chronic conditions in order to have some control of their care [20, 21].. Involvement in decision-making is a positive step in improving care of patients and has implication in self-management and adherence to treatment plans as these are mutually agreed on [22, 23]. A study conducted in Ivory Coast reported patients requesting to be involved in decision making regarding their treatment as a way of improving their care and reducing the burden of disease [24].

Some of the participants reported no participation in decision making in this study and others showed minimal involvement, leaving all decision regarding their treatment to be made by health care providers reflecting findings from other studies in the region [6, 25]. Several factors were reported by patients to be reasons for this behavior, one of them being belief that health care providers know what is good for patients and they should be making all the decisions. Other patients drew the analog of traditional healers who makes all the decision regarding their healing. Leaving decision making to providers may be considered as autonomy in passive mode which undermines patient rights. Educating and empowering patients on their rights and roles in decision-making will improve their participation leading to improved outcome.

Participants in this study reported scarcity of materials in the clinic especially decision making aids. Decisions aids are tools which are utilized to educate patients about their conditions, complications, effects of treatment, alternative treatment regimes, and other aspects of care, some of the tools are prepared to be used out of clinic settings [26]. In this study participants reported to be using materials available in the clinic that were necessary for health education. However, these materials were not sufficient to support decision making during consultation. Effectiveness of decisions aids depend largely on how the materials were prepared, the nature of contents and how they are utilized [27]. Participants indicated aids available in the clinic to be a good source of knowledge, however, it was difficult for them to link their use and shared decision-making. Studies have portrayed that decision aids are not only preferred as knowledge tools but also may facilitate shared decision-making [28, 29].

Beliefs and values, time and literacy or education level were reported as barriers to involvement in decision making in this study. Some patients reported to embrace the culture that does not allow individuals to meddle in decisions when consulting people of higher status for instance leaders. This is in line with many African societies, which observe cultural values, and beliefs that allow people with high status like doctors and leaders to decide for small profiled people [6, 25]. A study done in Malawi indicated that even decision to go to hospital is made by key people rather than patients themselves [30], implying minimal or no engagement in decisions when they reach the hospitals.

Limited consultation time is another important barrier to decision making reported by participants, time as a necessary resource in shared decision making and has been reported in many studies from resource limited settings which have high patients to provider ratio [31, 32]. It is obvious that some patients experienced minimal participation because of time constraints, due to patients load and other multiple tasks assigned to the attending medical specialists.

Literacy level of patients was reported by health care providers as one of the barriers in engaging patients, although this phenomenon is common in many resource limited settings, it should not be used to deny patients their right in making decisions [6]. A study done in Rwanda indicated that people with limited literacy were reliant on providers for decision making [25]. Health care providers are expected to customize their communication to suit the education need of patients to ensure understanding of available options, inform and empower patients in making appropriate choices.

Despite the important findings of this study, there are several limitations, which we need to acknowledge including the small number of health care providers interviewed in this study, which might have not provided saturation during data collection and findings obtained in this study were not confirmed by participants. Furthermore, this study did not explore the actual process of decision-making.

## Conclusion

This study has documented experience of healthcare providers and patients in shared decision-making at Muhimbili National Hospital (MNH) diabetic clinic through engagement of patients in making decisions.. Shared-decision making was believed to be vital for good outcome in long-term management of chronic conditions. However, the effective implementation of shared decision making have been hampered by scarcity of resources, patients’ literacy and belief. Despite these important findings this study did not assess the actual decision-making practice, which could be addressed in future studies.

From the findings of this study, it is important for diabetic clinic at Muhimbili National Hospital to devise mechanisms, which will empower patients to fully engage in shared decision-making. This engagement will improve self-management, adherence to care and ultimately improve overall outcome of care.

## Supplementary Information

**Additional file 1.**

**Additional file 2.**

## Data Availability

Data collected and used in this manuscript are available from corresponding author on reasonable request.

## Abbreviations

MNH: Muhimbili national hospital
SDM: Shared decision-making
