# Supplementary material for: Shared decision-making between health care providers and patients at a tertiary hospital diabetic Clinic in Tanzania
Source: BMC Health Serv Res. 2021 Jan 4;21:8. doi: 10.1186/s12913-020-06041-4 (PMC7780625; doi:10.1186/s12913-020-06041-4)
Supplement: Supplementary file 1 — Additional file 1. [file 12913_2020_6041_MOESM1_ESM.docx]

**Supplementary material - 1**

**In-Depth Interview Guide for Health Care Provider**

**
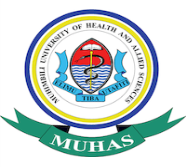
**

This is an in-depth Interview Guide for exploring healthcare providers’ and socio-cultural related factors that influence diabetic patients’ participation to shared decision making and the decisions aids used.

**Part I: Introduction**

Your responses are and will be accepted without reservations, so kindly feel free. I would like to remind you that, this interview will be recorded as for easy remembering of what you shared, and it will take about 30-45 minutes’ duration.

**Part II: Demographic Information**

If you don’t mind, kindly tell me about your

1. Age
2. Sex
3. Male
4. Female
5. Education level____________________________
6. Have you undergone any training on ethics/medical ethics?

**Part III: General Information:**

1. What influenced you to be a healthcare provider in particular to diabetic patients?
2. What is your experience in taking care of diabetic patients?
3. What are your roles in this department?

(**Probe:** length held position, professional background)

**Part IV: General questions**

1. Based on your roles and experience, kindly tell me what do you understand about diabetic participation to shared decision making? (**Probe:** perception, skills and understanding)
2. What do you understand about decisions aids? (**Probe:** skills, training, experience)
3. Can you tell me more about the hospital or department guidelines for ensuring shared decision making and the use of decision aids?

**Part V: Other related Factors That Influence Diabetic Patients’ Participation to Shared Decision Making:**

1. What are the related factors as you think that influence diabetic patients to participate into shared decision making?(**Probe:** skills, values and beliefs, healthcare-patient relationship, communication skills, institution support )

**Part VI: Significance of Shared Decision Making and the Use of Decisions Aids**

1. Kindly, explain the importance of engaging diabetic patients to shared decision making? (**Probe**: benefits to patients, family members, healthcare providers, department/hospital)
2. Kindly explain the importance of using decision aids in executing shared decision making? (**probe**: benefits to patients, healthcare provider, family member, institution)

**Part VII: Challenges Encountered in Shared Decision Making with Diabetic Patients**

1. What are the challenges encountered in the exercise of shared decision making with diabetic patients? (**Probe:** family pressure, guidelines, language, inferiority complex, time, professionalism experience, decisions aids)

**Part VIII: Strategies to Improve the Execution of Shared Decision Making**

1. According to the challenges against shared decision making what are your opinions on how best to implement shared decision making? (**Probe**: At the Individual level, at the Department/hospital level, at the National level)
2. What do you think are the viable decisions aids in our setting? (**Probe**: leaflets, booklets, internet or online application, posters, training programs, mass media)

**Part IX: Ending:**

I really appreciate the time you took for this interview. Do you have any other additional information that you find important and would like to share with me pertaining to the study?

I beg your cooperation once again when I will need more clarification about our interview.

**Thank you very much for your cooperation**
