# Supplementary material for: Shared decision-making between health care providers and patients at a tertiary hospital diabetic Clinic in Tanzania
Source: BMC Health Serv Res. 2021 Jan 4;21:8. doi: 10.1186/s12913-020-06041-4 (PMC7780625; doi:10.1186/s12913-020-06041-4)
Supplement: Supplementary file 2 — Additional file 2. [file 12913_2020_6041_MOESM2_ESM.docx]

**Supplementary material - 2**

**In-depth Interview Guide for Patients**

**
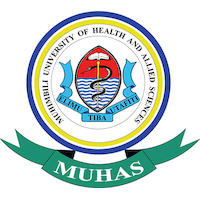
**

This is a patient’s in-depth Interview Guide for finding out patient’s related factors that influence diabetic participation to shared decision making and the decisions aids used at Muhimbili National Hospital.

**Part I: Introduction**

I have selected you among others hoping that you have rich information and you are capable of responding my questions. Your responses are and will be accepted without reservations, so kindly feel free. I would like also to remind you that, this interview will be recorded as for easy remembering of what you shared, and it will take about 30-45 minutes’ duration.

**Part II: Demographic Information**

May you kindly tell me about your

1. Age:
2. Sex:
3. Male
4. Female
5. Marital status
6. Single
7. Married
8. Occupation_________________________________
9. Education level______________________________

**PART III: General Question**

1. What do you understand about patients’ participation to shared decision making and the use of decisions aids?

**PART IV: Specific Questions**

1. What are the decisions aids used by healthcare providers to provide medical information? (Probes: leaflets, posters, face to face interview, mass media, internet) what decisions aids you like?
2. What do you think are the importance of engaging a diabetic patient to shared decision making? (Probes: for a patient, healthcare provider, family relative)
3. Do you think a patient’s relative has to be involved in decision making regarding his or her relative treatments? Why?
4. How far do you know about patients’ rights? (Probes: treatment guideline, informed consent form, where to claim your rights)
5. In your experience in health services what do you think are the challenges that hinder patients’ participation to SDM? (Probes: beliefs and values, inferiority complex, patient-healthcare relationship, family pressure, health literacy, time)
6. What are your suggestions on the diabetic patient to participate in decision making regarding his or her treatment?

**PART V: ENDING**

Do you have any other additional information concerning the study that you find important and would like to share with me pertaining to patients’ participation to shared decision making and the use of decision aids?

I beg your cooperation once again when I will need more clarification about our interview.

**Thank you very much for your cooperation**
